# Evaluation of the Sedative Effect of Limonene to Reduce Stress During Transportation of Gilthead Seabream (*Sparus aurata*)

**DOI:** 10.3390/biology14020115

**Published:** 2025-01-23

**Authors:** Paula Simó-Mirabet, Anyell Caderno, María José Flores-Llano, Elisia Gomes da Silva, William Schoenau, Bernardo Baldisserotto, Gonzalo Martínez-Rodríguez, Juan Miguel Mancera, Juan Antonio Martos-Sitcha

**Affiliations:** 1 Department of Biology, Faculty of Marine and Environmental Sciences, Instituto Universitario de Investigación Marina (INMAR), Campus de Excelencia Internacional del Mar (CEI·MAR), University of Cádiz, 11519 Puerto Real, Spain; anyell.caderno@uca.es (A.C.); mariajoseflores1998@gmail.com (M.J.F.-L.); juanmiguel.mancera@uca.es (J.M.M.); juanantonio.sitcha@uca.es (J.A.M.-S.); 2 Department of Physiology and Pharmacology, Universidade Federal de Santa Maria, Santa Maria 97105-900, RS, Brazil; elisia.silva@ufsm.br (E.G.d.S.); william.schoenau@ufsm.br (W.S.); bernardo.baldisserotto@ufsm.br (B.B.); 3 Department of Marine Biology and Aquaculture, Institute of Marine Sciences of Andalusia (ICMAN), Spanish National Research Council (CSIC), 11519 Puerto Real, Spain; gonzalo.martinez@csic.es

**Keywords:** aquaculture procedures, essential oils, fish transportation, HPI axis, welfare

## Abstract

Reducing stress during common aquaculture practices such as transportation is essential for improving animal welfare and aquaculture productivity. This study assessed the sedative effect of limonene, a key component of citrus essential oils, in a 6 h transport simulation of gilthead seabream (*Sparus aurata*), including four experimental groups: (i) non-transported, (ii) control (without treatment), (iii) ethanol (with ethanol), and (iv) limonene (30 µL/L dissolved in ethanol). After transport, the slightly reduced O_2_ consumption observed in the limonene group could be indicative of a reduced metabolism supported by a significant increase of chloride levels concerning other transported groups and an increase of sodium ions in fish treated with limonene. The increase of cortisol in transported groups supports its use as a good marker of acute-stress response in fish transport, showing a smaller increase in fish treated with limonene. This fact, together with the significantly reduced glucose and increased triglycerides in plasma, and the reduced expression of stress-related genes in the head kidney, may suggest a more relaxed state in fish treated with limonene. Since the sedative benefits of limonene were partly masked by the negative effects of ethanol, finding an alternative solvent could enhance its potential as a sedative for fish transport.

## Simple Summary

Common aquaculture practices such as fish transport can induce stress. With interest in animal welfare increasing among consumers as well as for the industry, the main objective of this study was to evaluate the sedative action of limonene to reduce stress during fish transportation in an important commercial species such as *Sparus aurata*. After the optimal dose of limonene was determined through measurements of the reduction in respiratory rate, fish transported for 6 h with the sedative showed higher water oxygen levels together with a more homeostatic state, judged by lower changes in osmolality or stress parameters (plasma cortisol and glucose and gene expression of stress biomarkers in the head kidney), than fish transported without limonene. Limonene-treated fish also presented a reduced metabolism, which may suggest a more relaxed state. However, the sedative benefits of limonene were partly masked by the negative effects of its solvent (ethanol); therefore, finding an alternative could enhance the potential of limonene as a sedative for fish transport. The findings of this research could help reduce fish stress derived from aquaculture practices, benefiting both fish and the industry.

## 1. Introduction

Aquaculture practices often expose fish to several stressors, which may activate the stress system, decreasing welfare status and negatively affecting several physiological processes, such as growth, reproduction, or immune response [1,2]. Currently, the transport of live fish is a common activity in the industry for various purposes, such as the carriage of fry from hatcheries to growth/fattening sites, the shipping of wild animals to aquaculture facilities, and the release of juveniles for stocking programs, public aquaria, the ornamental fish industry, or even for research [3]. Moreover, the increased demand for value-added products has also affected aquatic products and, according to this, marketing live fish provides a freshness guarantee, high quality for consumers, and higher price realization than the sale of fresh, chilled, or frozen goods (reviewed in [4]).

Transporting live fish includes several sources of stress, such as handling, overcrowding, changes in water conditions (temperature, oxygen, salinity, or waste substances), external stressors (e.g., vibrations) [5], or even a waterless transport in the case of flatfish [6]. This situation can alter fish homeostasis and stimulate the hypothalamus-pituitary–interrenal axis (HPI), generating diverse physiological and metabolic responses [7,8,9]. In addition, in a closed environment, the resulting metabolic waste excreted by fish is accumulated, affecting water quality parameters (i.e., pH, ammonia, carbon dioxide, and nitrite) that can compromise fish physiological functions and health. Therefore, it is necessary to improve transport conditions, creating reduced-stress environments to ensure animal welfare, which could decrease diseases and mortalities associated with these practices, resulting in better yields for farmers and a better quality of the final product.

In this sense, anaesthetics prevent or reduce the stress response, metabolic rate, and oxygen demand [10]. Thus, their application at a sedative concentration before or during transport may be a key tool for reducing stress and improving fish health and welfare [11,12]. However, some of the most used anaesthetics (i.e., MS-222, etomidate, quinaldine sulphate, benzocaine, phenoxyethanol, and clove oil) have been shown to activate the stress response in fish by themselves. Some of the side effects of these chemicals are physiological, such as changes in mucus secretion, gill irritation, and liver metabolism [13,14,15,16,17,18], but some are also associated with behavioural responses, such as aversion [19]. These studies show that anaesthetics have benefits and drawbacks; therefore, searching for compounds with minor or non-negative effects on fish, the environment, and human health is necessary. All this, together with a growing interest in the search for healthy food free of synthetic compounds, has made the use of anaesthetics of plant origin an option for the industry because they are economically viable and have fewer side effects than synthetic ones [9,20].

Essential oils (EOs) are a natural blend of volatile, lipophilic, and odoriferous substances obtained from aromatic plants [21] which may contain from 20 to 100 components from different chemical classes, predominantly terpenes [22]. EOs are distinguished by two or three major constituents (20–95%), other secondary components (1–20%), and trace components (<1%) [9]. However, the chemical composition varies according to plant species, part of the plant, ontogeny, and environmental conditions (e.g., climate, soil, season, etc.), as well as the extraction methods [23,24,25]. Interestingly, some EOs and their active compounds have antibacterial, antifungal, insecticide, antiparasitic, antioxidant, sedative, and anaesthetic properties. In addition, their use is increasing in aquaculture practices to facilitate handling, tagging, transport, vaccination, inducing laying, blood extractions, dissections, or surgery [9,26,27,28]. Most of these beneficial properties have also been found in EOs which contain limonene as the main or isolated compound [29,30,31,32,33,34]. Limonene belongs to a group of compounds known as monoterpenes, being the predominant constituent of citrus EOs. In addition to the above-presented benefits, different studies have demonstrated consistent anxiolytic effects in several behavioural tests in mice [35,36,37], and a reduction in depression-like behaviour in rats [38]. Remarkably, many studies have demonstrated that EOs with limonene are good anaesthetics for fish [39,40,41] and have likewise been shown to have sedative and anxiolytic effects on zebrafish [42]. Thus, the beneficial properties of limonene, as well as its natural origin, make it interesting to test this substance as a sedative for fish. However, the efficacy of EOs for reducing stress depends on their chemistry composition, concentration, or plant chemotype [41], as well as the species and size of fish used [27]. Thus, studying the isolated active compound of these EOs would help to establish and control their effects.

With increasing public concern for animal welfare, and its growing importance for fish farmers from the perspective of improving standards and the quality of fish products, welfare issues should be addressed to improve and reduce stress in aquaculture practices such as fish transport. In this kind of procedure, the addition of sedatives to the water can help reduce the negative consequences of transport on fish physiology and welfare. Furthermore, the use of suitable natural products as sedatives, such as limonene, would contribute to higher ecological and environmentally friendly aquaculture [9]. In this sense, few studies address possible solutions for reducing the negative effects of transport on fish, such as the application of natural sedatives to transport water, with many of them focusing on freshwater teleosts (reviewed by [3]). Therefore, this study aimed to evaluate the effectiveness of limonene, one of the main components of EOs derived from citrus plants, in reducing stress responses during the transport of a species of great commercial interest in marine aquaculture such as the gilthead seabream (*Sparus aurata*). Addressing welfare issues is in line with the improvement of production performance and the sustainability of the industry.

## 2. Materials and Methods

### 2.1. Ethics

This experiment was carried out following the guidelines for experimental procedures in animal research of the Ethics and Animal Welfare Committee of the University of Cadiz, according to Spanish laws (RD 53/2013 and RD 118/2021) and European Union legislation (2010/63/UE). The Ethical Committee from the Autonomous Andalusian Government approved the experiments (Junta de Andalucía reference number 3/11/2021/173).

### 2.2. Determination of the Optimal Sedative Concentration of Limonene

The sedative effects of limonene in gilthead seabream juveniles were evaluated to determine the most effective and safest concentration range to use for short-distance transportation (6 h). For that, animals (100 g mean body weight) from the experimental facilities of *Servicios Centrales de Investigación en Cultivos Marinos* (SCI-CM, CASEM, University of Cadiz, Puerto Real, Cádiz, Spain; Facilities for Breeding, Supplying and Users of Experimental Animals; Spanish Operational Code REGA ES11028000312) were exposed to four different concentrations of limonene (LMN10, 20, 30, and 40 μL/L) based on previous studies performed on freshwater species (B. Baldisserotto, personal communication). The present experiment was performed with commercial R-(+)-Limonene 97% (Sigma-Aldrich, Sant Louis, MO, USA, Ref. 183164), obtained from isolating and purifying the active compounds present in the EOs to achieve a more reproducible (i.e., more stable) composition for testing and to avoid the undesired effects of other compounds. Due to its hydrophobic nature, it was necessary to pre-solubilize limonene with ethanol (1:9) in a glass beaker, where seawater was added to the mix until completely dissolved. Finally, this blend was added to each tank before starting the experiment. Negative controls were performed with ethanol (E) at the same amount (3.6 mL) used to dilute the highest limonene concentration. Also, a positive control without treatment was assessed (CTRL). To determine the optimal concentration of limonene, eight fish per treatment (n = 48) were introduced in pairs in 10-L aquaria with aeration and were monitored for respiratory activity (opercular movement rate), assessed by counting the opercular beats for 15 s after 6, 10, and 20 min of compound exposure. In addition, to know if the fish had achieved a sedative state, we evaluated the reactivity to external stimuli at each time point, knocking the external part of the aquaria, touching the anteroposterior dorsal side of the animal with a glass tube, and pinching the caudal peduncle of the fish. The same person conducted the measures to avoid subjective assessments. After 20 min of exposure, they were removed and transferred to a tank without treatment to monitor the recovery time, which was complete when fish demonstrated normal swimming and responded to external stimuli. The induction and recovery times were measured and registered with a digital timer. Considering deep sedation as the ideal level for transporting fish, based on stages of anaesthesia in fish described by McFarland (1959) [43], we established the induced deep sedation time at 3 min, while the recovery time was set at 5 min. Finally, since all treatments showed sedation and recovery times within the established values, in which fish can maintain equilibrium without responding to external stimuli, we decided to evaluate the sedative potential of each concentration according to a more reproducible and objective measurement, such as changes in respiratory rate. After their recovery, the animals were returned to their initial tanks, provided with clean and oxygenated water, and were not used for other tests. No mortality was observed after 48 h.

### 2.3. Behaviour Analyses with Video Recording

Fish movements were captured through video recording to evaluate the behavioural responses to exposure to different limonene concentrations. Ten fish from each of the following treatments were evaluated: (i) CTRL, control group without treatment; (ii) E, with the same amount of ethanol used to pre-dilute limonene; (iii) LMN10, with a final concentration of 10 μL/L limonene previously pre-diluted as described above; (iv) LMN20, with a final concentration of 20 μL/L limonene previously pre-diluted as described above; and (v) LMN30, with a final concentration of 30 μL/L limonene previously pre-diluted as described above. Briefly, aquarium tanks were prepared with 10 L of seawater, and the limonene was added as explained above. Then fish were introduced individually into the tank and recorded for 20 min after treatment exposure using a digital camera (Model: Nikon D5100) on a tripod. The recordings were carried out at the same time of day with natural light, and the tanks were provided with a black background to avoid light reflections. All recorded videos were analysed through direct visualization by the same person just after treatment exposure (T0), as well as after 6 (T6), 10 (T10), and 20 (T20) min. The following behaviour parameters were studied: time at the bottom (s), time leaning down/up (s), freezing time (s), distance travelled (cm), mean speed (m/s), and the number of complete turns and crossings (Table 1).

### 2.4. Short-Distance Transport Simulation and Sampling

Gilthead seabream juveniles from stock tanks (10 m^3^ volume) from the experimental facilities of *Servicios Centrales de Investigación en Cultivos Marinos* were distributed between 12 experimental tanks (500 L volume, 4 per experimental condition; density: 3 kg/m^3^) and were acclimated for two weeks before the transport simulation to avoid any undesirable effects during transport and posterior analyses, and also to monitor the recovery of transported fish easily. Throughout this period, fish were maintained under controlled environmental conditions of salinity (37 ‰), temperature (18.5 °C), and photoperiod (10L:14D), and fed to visual satiety with a commercial aquafeed (EFICO Plus 805; BioMar, Dueñas, España). Then, overnight-fasted fish were placed in plastic bags (3 fish/bag and 4 replicates per treatment) saturated with oxygen at a density of 30 kg/m^3^ and transported for 6 h by a transport van. Fish were introduced into the plastic bags at intervals of 15–20 min to ensure the same transport time in each one and to facilitate the biometric and tissue sampling. The initial water conditions were 37 ‰ salinity and 18.5 °C. Then, the transported fish constituted the following experimental groups: (i) control (CTRL), without treatment; (ii) ethanol (E), with the same amount of ethanol used to dilute limonene; and (iii) limonene (LMN30), with 30 µL/L limonene dissolved in 9 parts of ethanol. One hour before finishing transport, 12 fish from the initial tank were sampled in the same conditions (overnight fasted), constituting the pre-transport group (PRE), without transportation. After 6 h of transport, fish were sampled in the same order in which they were placed in the transport bags. First, water temperature, oxygen, and pH were measured from the bags and extra samples were collected in 50 mL falcon tubes for posterior analyses. Then, 12 fish from each experimental group (3 fish/plastic bag) were anaesthetized with a lethal concentration of 2-phenoxyethanol (1 mL/L sea water), and biometric and tissue samples were taken (blood, liver, and head kidney). Blood was drawn from the caudal vessels with heparinized syringes and centrifuged at 13,000× *g* for 20 min at 4 °C to acquire plasma. Before centrifugation, one aliquot was collected in haematocrit tubes and centrifuged at 13,000× *g* for 5 min. Samples for metabolic analyses were saved at −80 °C, while samples for molecular biology were introduced in 10 volumes of RNAlater Stabilising Solution and were maintained for 24 h at 4 °C and then frozen at −20 °C until further analyses.

### 2.5. Analysis

#### 2.5.1. Water Parameters

Water ammonium, nitrite, and nitrate levels were measured using handheld checkers (models: Ammonia Checker^®^ HC- HI784, Nitrate Checker^®^ HC- HI781, and Ultra Low Range Nitrite Checker- HI-764) from HANNA instruments^®^ (Eibar, Spain). Briefly, ammonium measurement was based on an adaptation of the Salicylate Method, where the reaction between ammonia and ammonium and the reagent causes a blue-green tint in the sample. On the other hand, the Beer–Lambert principle was used to determine the concentration of nitrate colourimetrically. Finally, nitrite levels were obtained from the colourimetric reaction based on an adaptation of the EPA Diazotization Method 354.1. In all cases, the protocol consisted of a first read of the water sample (considered blank), and then the addition of the reactive provided in the commercial kit, following the manufacturer’s instructions.

#### 2.5.2. Osmolality and Ion Levels in Plasma and Water

Osmolality was measured with the Fiske^®^ Micro-Osmometer, Model 210 (Advanced Instruments, Norwood, MA, USA). Ion levels were determined with commercial kits (Potassium-LQ, Ref. 1001397; and Sodium-LQ, Ref. 1001387; SpinReact S.A., St. Esteve d′en Bas, Girona, Spain) adapted to 96-well microplates following the manufacturer’s instructions.

#### 2.5.3. Cortisol in Plasma

Plasma cortisol was measured using the Enzyme Immunoassay Kit (Arbor Assays, Ann Arbor, MI, USA, K003-H1W), according to the manufacturer’s instructions. Briefly, plasma samples were treated with 2 volumes of dissociation reagent and then diluted with assay buffer at the expected measurable range. Samples and standards were plated in duplicate, then the specific monoclonal antibody and cortisol-peroxidase conjugate were added, and the plate was incubated with strong agitation (850 rpm) at 24 °C for 1 h in the shaker-thermostat Sky Line (Elmi, Newbury Park, CA, USA). Then, the plate was washed with the Wash buffer supplied by the kit, and the 3,3′,5,5′-tetramethylbenzidine (TMB) substrate was added and incubated for 30 min at 24 °C without agitation to produce a colourimetric reaction with the bound cortisol-peroxidase conjugate. The reaction was stopped with the addition of Stop solution, and the plate was read at 450 nm.

#### 2.5.4. Metabolites in Plasma and Liver

Liver samples were mechanically homogenized with the T25 digital Ultra-Turrax^®^ (IKA-Werke, Staufen, Germany) with the dispersing tool S25N-10G in 7.5 volumes of ice-cold 0.6 N HClO_4_ and then neutralized using 1 M KCO_3_. Before centrifugation, an aliquot from all homogenates was frozen at −80 °C for posterior analyses of triglycerides (TAG). The rest of the homogenate was centrifuged at 4000× *g*, 4 °C for 30 min and the resulting supernatants were used to determine the rest of metabolites. Tissue glycogen concentration was quantified using the method described by Keppler and Decker (1974) [44], in which glucose obtained after glycogen breakdown with amyloglucosidase (Ref. A7420, Sigma-Aldrich) was determined with the commercial kit as described above. Plasma and liver metabolites were measured spectrophotometrically using commercial kits (SpinReact S.A., St. Esteve d′en Bas, Girona, Spain) adapted to 96-well microplates, and include the levels of glucose (Ref. 1001200), lactate (Ref. 1001330), cholesterol (Ref. 41021), and triglycerides (Ref. 1001311). Plasma total protein concentration was determined using the commercial BCA kit based on the bicinchoninic acid method (BCA™ Protein assay kit, Pierce, Rockford, IL, USA). All assays were performed with a PowerWave™ 340 microplate spectrophotometer (BioTek Instruments, Winooski, VT, USA) and KC Junior Software 1.41 for Microsoft^®^ Windows.

#### 2.5.5. Gene Expression in the Head Kidney

Head kidney portions were homogenized using the Ultra-Turrax^®^ T25 with the dispersing tool S25N−8G (IKA-Werke, Staufen, Germany). Total RNA from head kidney samples was isolated with the NucleoSpin^®^ RNA kit (Macherey-Nagel, Madrid, Spain). RNA quality was determined using a 2100 Bioanalyzer (Agilent Technologies, Santa Clara, CA, USA) and total RNA was quantified with Qubit™ RNA Broad Range Assay Kit by the Qubit^®^ 2.0 Fluorimeter (Thermo Fisher Scientific, Madrid, Spain). Only samples with RNA Integrity Numbers > 8.0 were used for gene expression analyses. For the cDNA synthesis, 500 ng of RNA from each sample was reverse transcribed in a 20 μL reaction volume using a qScript^™^ cDNA Synthesis kit (Quanta BioSciences^™^, Beverly, MA, USA) in a Mastercycler^®^ proS (Eppendorf AG, Hamburg, Germany). The cDNAs were diluted 1/10 with 10 mM Tris 0.1 mM EDTA pH 8.0, obtaining a final concentration of 2.5 ng/µL. Before gene expression analyses, a calibrator sample was used to determine the optimum quantitative PCR (qPCR) conditions for each gene. For this, a pool of cDNAs from all samples was used, and six 1/10 serial dilutions (from 10 ng to 100 fg) were made to obtain calibration curves for each pair of primers. Additionally, control reactions with RNase-free water (NTC) and RNA (NRT) were included in the analysis to ensure the absence of primer-dimers and genomic DNA contamination, respectively.

Using Hard-Shell^®^, white well/white shell, low-profile, thin-wall, 96-well, skirted PCR plates covered with Microseal^®^ B Adhesive Seals (BioRad, Hercules, CA, USA), each reaction was performed in duplicate with 0.5 μL (final concentration 200 nM) of each pair of primers (Table 2), 5 μL PerfeCTa™ SYBR^®^ Green FastMix™ (Quanta Bio, Beverly, MA, USA), and 4 μL of cDNA (10 ng). The qPCR reactions were performed in a CFX Connect™ Real-Time PCR System (Bio-Rad Laboratories, Hercules, CA, USA) as follows: an initial denaturation and polymerase activation at 95 °C for 10 min, followed by 40 cycles of denaturation for 15 s at 95 °C, annealing and extension at 60 °C for 30 s, and finishing with a melting curve from 60 °C to 95 °C, increasing by 0.5 °C every 5 s. Melting curves were used to ensure that only a single PCR product was amplified and to verify the absence of primer-dimer artifacts. Relative gene expressions were analysed with CFX Manager™ software version 2.3 (Bio-Rad Laboratories, Hercules, CA, USA), using the ΔΔC_T_ method [45], and corrected for efficiencies [46]. To normalize the results, actin beta 1 (*actb1*) and eukaryotic elongation factor 1 alpha (*eef1a*) were chosen as internal reference genes. These two reference genes were selected owing to their low variability in the present experiment (CV = 0.1044, M = 0.3018), complying with the requirements established by the GenNorm Target Stability Value (CV < 0.25 and M < 0.5).

### 2.6. Statistics

All results are expressed as the mean ± SEM (standard error of the mean) of 8 (opercular movements), 10 (behavioural analyses), and 12 (physiological and molecular parameters) fish. All data were checked for normality and homogeneity of variance using Shapiro–Wilk and Brown–Forsythe tests, respectively, with *p* < 0.05. Two-way analysis of variance, followed by Tukey’s test, was performed to assess the effect of treatment (CTRL, E, LMN20, and LMN30) over time (T0, T6, T10, and T20) in several behavioural parameters measured in video analyses, as well as one-way ANOVA (*p* < 0.05) followed by Tukey’s test to determine the differences among treatments at each time. Likewise, in the determination of the optimal sedative concentration of limonene (opercular movements) and parameters measured after the transport experiment, differences among treatments in all parameters were analysed via one-way analysis of variance (ANOVA, *p* < 0.05) followed by Tukey’s test. The software package GraphPad Prism 9.0 (GraphPad Software, Inc., San Diego, CA, USA) was used for statistical analyses and the preparation of figures.

## 3. Results

**Figure 1:**
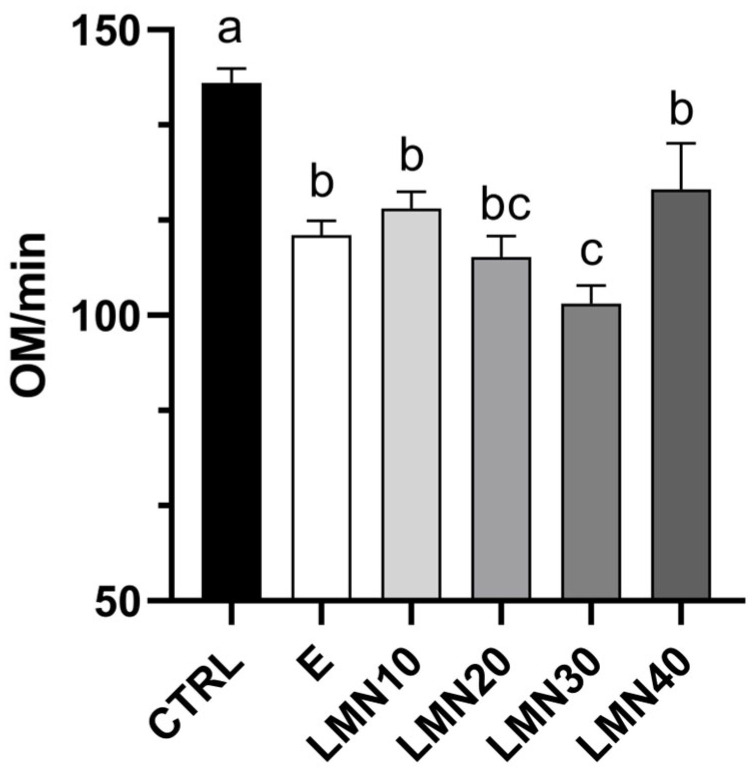
Respiratory frequency measured as opercular movements (OM)/min in gilthead seabream after repeated measures over 20 min. Transported fish without treatment (CTRL), with ethanol (E), or with limonene (LMN) at different concentrations (10, 20, 30, and 40 µL/L). Data are the mean ± SEM of 8 fish. Different letters indicate significant differences among treatments (one-way ANOVA followed by Tukey’s test *p* < 0.05).

The preliminary test to determine the best concentration to use as a sedative during short transportation showed, in all cases, induction and recovery times inside the parameters established in the Material and Methods section. Furthermore, observations related to measurements of reactions to external stimuli (reduced response) and the behavioural analyses (e.g., more exploratory behaviour, as discussed later) suggested that the concentration of 30 µL/L was the most suitable. This was corroborated by a more objective measurement based on counting opercular movements/min, which demonstrated a lower respiratory frequency. For all these reasons, the concentration of 30 µL/L was used to evaluate the efficiency of limonene in the transport of *Sparus aurata* (Figure 1).

Video analyses revealed that seven out of the eight behavioural parameters measured were dependent on exposure time, of which five were also dependent on treatment, while only one showed an interaction (time *x* treatment) (Table 3). The time at the bottom decreased 10 min after fish were placed into the water with the sedative, returning to the initial values within 20 min, especially in the groups treated with limonene that rested for less time at the bottom. However, at the higher concentration of limonene (LMN30), this parameter showed differences at the beginning (T0), and the same trend was observed at 6 and 10 min, while the lowest concentration of sedative (LMN20) presented intermediate values. After 20 min, the values were similar among the different experimental groups. Other parameters, such as the time leaning down or the number of complete turns, diminished over time regardless of treatment. Interestingly, the time leaning up showed no differences among treatments or over time. On the other hand, after 6 min of exposure to the sedative, the time that fish stopped (freezing time) increased, with the highest values presented by the CTRL group while both limonene groups (LMN20 and LMN30) were the most active. Finally, the distance travelled decreased over time, except in the LMN30 group, with an increase within 20 min, which maintained the highest values throughout the trial. Regarding the treatments, the CTRL group travelled a shorter distance and at a lower speed, while the LMN30 group travelled greater distance at a higher speed. Finally, in the middle of the trial (T6 and T10), the LMN30 group presented more crossings than the rest of the experimental groups.

On the other hand, after the transport simulation, water acidification was observed in all groups (pH = 6.7) compared to the initial conditions (pH = 7.5). In addition, the transported fish presented a reduction in water O_2_ levels after 6 h of transport, without significant differences among groups. However, fish treated with limonene (LMN30) showed higher water O_2_ saturation values (84.8%) compared to the control (50.7%) and ethanol (60.8%) groups. Ammonium (≤0.004 ppm) and nitrites (<0.3 ppm) remained below toxic levels in all experimental groups. All water parameters are summarized in Table 4.

**Figure 2:**
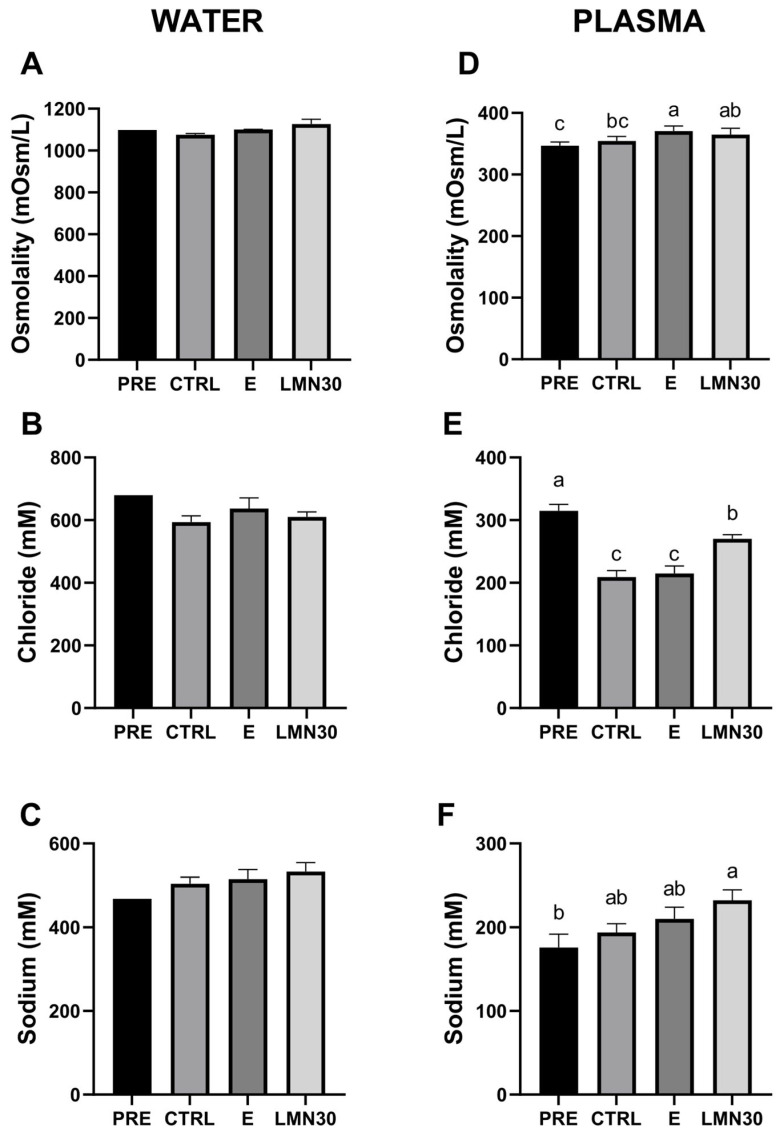
Osmolality (**A**,**D**), chloride (**B**,**E**), and sodium (**C**,**F**) levels in water and plasma of gilthead seabream after 6 h transport. Non-transported fish (PRE) and transported fish without treatment (CTRL), with ethanol (E), or limonene (LMN30). Data are the mean ± SEM of 4 plastic bags or 12 fish for water and plasma analyses, respectively. Different letters indicate significant differences among treatments (one-way ANOVA followed by Tukey’s test *p* < 0.05).

Likewise, the osmolality and chloride and sodium ions present in the water remained constant among groups (Figure 2A–C). However, a slight increase in plasma osmolality was observed in all transported fish, with the E group (Figure 2D) showing the highest values. Chloride ions diminished in the CTRL and E groups, while they were partially restored in the LMN30 group, resembling the values of the pre-transport group (Figure 2E). On the contrary, sodium ions increased in transported groups, reaching the highest values in fish treated with limonene (Figure 2F).

**Figure 3:**
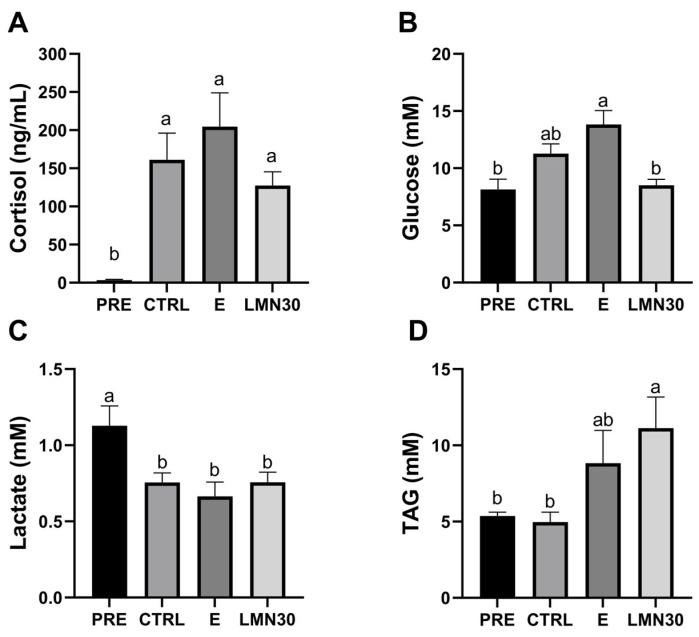
Plasma cortisol (**A**), glucose (**B**), lactate (**C**), and triglyceride (TAG) (**D**) levels of gilthead seabream after 6 h transport. Non-transported fish (PRE) and transported fish without treatment (CTRL), with ethanol (E), or limonene (LMN30). Data are the mean ± SEM of 12 fish. Different letters indicate significant differences among treatments (one-way ANOVA followed by Tukey’s test *p* < 0.05).

Regarding blood parameters, no changes in haematocrit levels were observed among transported groups, but a slight increase was detected compared to non-transported fish (Table 5). Cortisol increased greatly in all transported fish, with the lowest values presented by the limonene group. Plasma glucose increased in the CTRL and E groups, while fish treated with limonene did not show that increase. Lactate levels decreased after transport in all experimental groups, while TAG increased only in the E and LMN30 groups, with the highest values occurring in the latter (Figure 3). Plasma protein and cholesterol kept constant levels among different groups (Table 5). In the liver, no significant differences were detected among groups for any metabolite measured. Nevertheless, free glucose, lactate, and TAG levels tended to increase in transported fish regardless of the treatment tested (Table 5).

**Figure 4:**
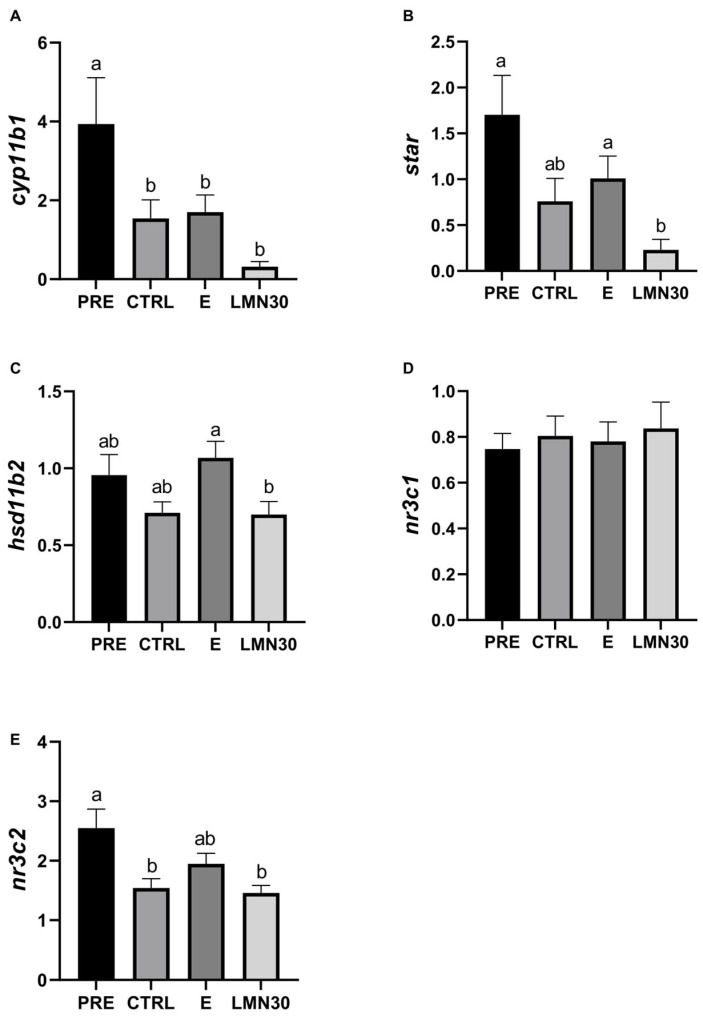
Expression levels of several stress-related genes in the head kidney of gilthead seabream after 6 h transport: (**A**) *cyp11b1*; (**B**) *star*; (**C**) *hsd11b2*; (**D**) *nr3c1*; and (**E**) *nr3c2*. Non-transported fish (PRE) and transported fish without treatment (CTRL), with ethanol (E), or limonene (LMN30). Data are the mean ± SEM of 12 fish. Different letters indicate significant differences among treatments (one-way ANOVA followed by Tukey’s test *p* < 0.05).

Finally, four out of five stress-related genes (*cyp11b1*, *star*, *hsd11b2*, and *nr3c2*) measured in the head kidney showed similar gene expression patterns, exhibiting lower levels in transported fish. Specifically, the LMN30 group presented the lowest values for both the *cyp11b1* and *star* genes (Figure 4).

## 4. Discussion

The increasing public consciousness regarding the welfare of production animals poses a challenge for the aquaculture sector [47,48,49]. Indeed, stress caused by common aquaculture practices such as handling, transportation, crowding, and grading, among others, can compromise fish welfare, reducing profitability and leading to direct negative effects for aquaculture producers [9,50]. In view of this, and considering the increasing awareness of the *One Health* concept, the present work aims to improve and refine fish transport protocol, employing limonene, one of the main constituents of several essential oils, as a sedative [51].

According to recommendations for selecting the optimal concentration of a sedative for fish transport [52,53], we first established the best amount of limonene, set at 30 µL/L, to evaluate its possible use as a sedative in the transport of *S. aurata*. This was supported by a reduction in fish response to external stimuli (without loss of equilibrium) and respiratory frequency (measured as opercular movements/min). Indeed, fish under sedation reduced opercular movements compared to untreated fish, corroborating the state of light sedation achieved in this species, which may be useful for its application in transport procedures. Likewise, changes in behaviour are essential for assessing the welfare of farmed fish [54]. In fact, most of the behavioural parameters measured via video analysis were useful in determining the effect of limonene as a sedative. The exception was found for time-dependant parameters such as the number of complete turns and the time leaning down. The first one decreases over time (higher at the beginning and sustainably reducing after 6 min), probably as part of exploratory behaviour [55]. In addition, the lack of changes in the leaning up parameter may indicate an optimal oxygen saturation in the water, since fish respond to declining oxygen levels by ventilating at the water surface, a behaviour named “aquatic surface respiration” [56,57].

Furthermore, avoiding the water’s surface may be indicative of the lack of aversive behaviours caused by limonene, such as the scape-like reactions presented in fish exposed to anesthetic overdoses [58] or toxic compounds [59]. This could suggest that limonene did not generate a toxic reaction in the fish. Accordingly, exposure to novelty/unfamiliar environments elicits anxiety behavioural responses in different species, characterised by a reduction in time spent at the top of the tank (where dangers may appear), an increase in erratic movements, and freezing [60]. In the present experiment, fish treated with limonene spent less time at the bottom of the tank, especially during the first few minutes, when exploratory behaviour occurs, as well as spending less time immobile (freezing behaviour). Félix et al. (2021) [61] evaluated the potential use of different anaesthetics in Nile tilapia in a 6 h transport experiment in which fish with lower opercular rates and lower responses to external stimuli spent more time swimming (and were less inactive) and showed lessened stress-related responses, as occurred in the current experiment.

Fish exposed to unpredictable stressors presented less exploratory behaviour [62]. However, the higher number of crossings and the distances travelled at a greater speed by fish from the limonene groups (especially LMN30) are indicative of increased exploratory behaviour, suggesting a sedative or more relaxed state in the fish, provided by the limonene. The distance travelled decreased over time in all groups, but the highest values were observed in the LMN30 group, which could also be related to a more relaxed state and less anxiety about the novelty of the tank. It is important to note that, despite the constant trends observed in behavioural parameters, some of them could be affected by the lipid nature of limonene, with a possible unequal distribution into the water, despite the fact that the compound was completely dissolved in ethanol before its use [61]. In addition, Almeida et al. (2019) [63] described a reduction in swimming activity in *Serrasalmus eigenmanni* because of ethanol, which enhances the action of several GABA receptor subtypes, but this was a transient effect due to ethanol evaporation during the first 5 min. We probably did not see those effects due to the differences in video recording time (after 6 min) in our study. All this together could indicate that limonene provides an anxiolytic and sedative effect in *S. aurata*, as has been described in zebrafish [42], and therefore could be considered as a potential candidate for reducing stress responses during fish transportation, following one of our main objectives.

The deterioration of water quality parameters can be stressors per se that negatively affect fish welfare during transportation. After 6 h of transport simulation, water acidification occurred, which is a typical response in short-distance transports (<8 h) because of an increase in O_2_ consumption and the subsequent greater release of CO_2_ into the water [64]. A decrease in O_2_ levels was observed in all groups after transportation, although they remained at optimal values due to the water saturation before transportation. However, although it was not significant, the group transported with limonene presented higher O_2_ saturation compared to the CTRL and E groups, in agreement with the fact that EOs as sedatives improve or preserve the water quality [39]. Additionally, ammonium and nitrite levels were kept below levels considered toxic in all transported fish, contributing to maintaining fish welfare during transport.

The stressful conditions associated with fish transportation increase respiration rate, inducing a loss of ions in freshwater and water loss in saltwater and, consequently, an alteration in osmotic balance [34,65]. The resulting hydromineral disturbances are orchestrated by the increased permeability of the surface epithelia (i.e., the gills and skin) to water and ions due to enhanced circulating catecholamine levels, as the first step of the stress response activating the HPI cascade [66]. In this regard, there are several studies on freshwater fish species (reviewed in [9]); however, less is known about the effects of transport on marine fish. In the present experiment, osmolality and ion levels (sodium and chloride) in the water were unaltered. Conversely, plasma osmolality increased in fish transported with ethanol and limonene compared to non-transported fish, different from that which was described by Jerez-Cepa et al. (2019) [67], where no changes were observed in plasma osmolality in the same fish species after 6 h of transport but variations (decreasing) presented after 18 h of recovery. Regarding plasma ions, and similarly to what occurs in freshwater species, the gilthead seabream experienced a reduction in chloride levels after transport, which was less pronounced in the limonene group, allowing for a better osmotic balance in the treated fish. On the other hand, the significant increase of sodium ions in the plasma of fish treated with limonene (concerning non-transported fish) may be related to the more relaxed state of these fish, which consumed less O_2_, as judged by the highest values of this gas in the water after transport. It is known that hypoxia (i.e., low oxygen content) stimulates sodium intake in the erythrocytes (decreasing its presence in plasma) due to the increased activity of Na^+^/H^+^ transport [68,69], which supports this hypothesis.

Consequently, plasma osmolality enhancement observed in fish treated with ethanol and limonene may be due to alterations in plasma ion levels (see above), although other osmolites (i.e., metabolites) could be involved in plasma osmolality alterations. Sampaio and Freire et al. (2016) [50] point out the importance of evaluating plasma osmolality in fish transport, due to its implication in homeostasis and its relationship with cortisol. In this sense, different studies suggest the role of cortisol in regulating Na^+^ balance by suppressing ion loss [70,71,72,73]. This agrees with our results, since small or no changes were observed in the water Na^+^ levels, highlighting the importance of cortisol in the regulation of ionic balance. Indeed, the increase of cortisol levels in all transported groups supports the use of this hormone as a good marker of acute-stress responses during fish transport, although its increase may be a combination of both previous handling and subsequent confinement in plastic bags. Interestingly, the lower cortisol levels exhibited by fish treated with limonene compared with other transported fish, although not statistically significant, may indicate the effectiveness of this compound in reducing stress in fish during transport. This is supported by the demonstrated efficacy of the EO *Aloysia triphylla*, which has limonene as its main constituent, as a sedative for the transport of *Rhamdia quelen* [74], *Lophiosilurus alexandri* [39], and *Serrasalmus eigenmanni* [63]. However, the effect of limonene on reducing cortisol levels was not enough, due to the negative effects caused by ethanol; an increase in cortisol levels has also been described in zebrafish after acute exposure to ethanol [75], as well as in different studies with EOs [20,76]. However, it is important to note that ethanol does not have any anaesthetic or sedative effect on fish, highlighting that the sedative effects of limonene are due to its natural composition. Cortisol release in transported fish could be related to the energetic cost of osmoregulation, as well as to other processes imposed by the stress situation, as exhibited by the subsequent metabolic changes. Therefore, as part of the secondary responses to stress, the lack of changes in haematocrit levels would suggest that this variable is usually more indicative for evaluating long-distance transports (>8 h) [50].

In our experiment, glucose levels followed a similar pattern to those of cortisol. The hyperglycaemia observed in transported fish, with the highest values exhibited in fish treated with ethanol, corroborates the negative effects of this substance on the fish stress system. On the other hand, fish treated with limonene presented the lowest values of this metabolite, which are surprisingly similar to those of the pre-transport group, thus pointing out the power of this natural compound in the regulation of energy metabolites such as glucose. In general, during transport, plasma glucose enhancement is accompanied by a decrease in hepatic glycogen [77,78], which has not occurred in the present study, where hepatic metabolites did not show differences among groups. However, metabolic reorganisation/orchestration in response to energy demand after a stressful situation is highly variable among species, and also depends on the time for which the stressor is maintained. Lastly, lower levels of plasma lactate in transported fish indicate a reduction in metabolism consistent with a typical stress response during transport [50] and similar to those described by Jerez-Cepa et al. (2021) [12] in the same fish species. In the same way, the higher levels of TAG in the limonene group could indicate a reduction in metabolic functions, thus inferring a more relaxed state provided by the chemical rather than by the lipolytic action of cortisol for energy generation [79]. Furthermore, several studies have described the benefits of EOs as sedatives to reduce metabolic rates and stress responses during transportation, mainly in freshwater fish [3,80,81,82]; this is demonstrated herein for seawater species, namely, *S. aurata*.

Finally, since similar studies have not detected changes in the expression levels of key genes at the central level (i.e., brain and pituitary) [67,83], we only analysed the mRNA expression in the head kidney as the final target tissue of the HPI cascade. Changes in the expression of the mineralocorticoid receptor (*nr3c2*) suggest greater participation of this type of receptor in the head kidney to locally or paracellularly regulate stress responses in fish, induced by transport. In comparison, the overall lower expression and lack of changes of the glucocorticoid receptor (*nr3c1*) after transport are consistent with its lowest affinity for cortisol compared to the mineralocorticoid receptor [84]. In addition, genes related to steroid hormone production (*cyp11b1* and *star*) showed similar change patterns (lower levels in transported fish, especially in the limonene group) and, interestingly, followed a similar pattern to plasma cortisol levels in all transported groups. This good correlation could be due to the participation of Star as the rate-limiting step of steroid biosynthesis, transporting cholesterol to the inner mitochondrial membrane [85], and Cyp11b1 in the conversion of 11-deoxycortisol to cortisol [86]. However, mRNA expression is not always parallel to the release of cortisol, as described by the authors of [67], who found a high correlation between the *cyp11b1* and *star* genes and a lack of coincidence with the cortisol profile, as occurred in the present study for non-transported fish. The lower expression values of all these genes, together with higher levels of cortisol in transported fish compared to non-transported fish, may be the result of the exhaustion of the system after 6 h of transport and/or the activation of negative feedback by plasma cortisol. Accordingly, *hsd11b2* contributes to the negative feedback regulation of cortisol during stress [87], with more stable expression levels in general, and its similar pattern with cortisol in non-transported fish corroborates a decreased need to stop the stress response in the case of fish treated with limonene, since it was less pronounced.

## 5. Conclusions

In conclusion, our results demonstrate that limonene induces a reduction in respiratory frequency and the response to external stimuli, as well as producing behavioural changes (i.e., increased exploratory behaviour). These observations would suggest a more relaxed state. In addition, limonene appears to have positive effects on maintaining oxygen levels in the water and on osmotic balance, probably due to the slight reduction in overall metabolism. Another remarkable positive effect of limonene as a sedative for fish during transport is the reduction of stress-related markers in the plasma and head kidney. However, the benefits of limonene appear to be partially masked by the negative effects of ethanol on several of the parameters measured. Therefore, it would be interesting to continue the search for other methods for its pre-dilution (e.g., nanoemulsions) to improve the effects of limonene, since this compound could have a great potential to reduce stress and could, therefore, improve animal handling and welfare, becoming a promising sedative for the transport of live fish.

## Figures and Tables

**Table 1:** Behavioural parameters assessed through video analyses of gilthead seabream juveniles in response to different limonene concentrations.

Behavioural Parameters	Definition
Time at the bottom	Time spent in the lower half of the aquaria
Time leaning down	Time in which the fish leaned with the head down at 45 degrees or more
Time leaning up	Time in which the fish leaned with the head up at 45 degrees or more
Freezing time	Time in which the fish did not move the body, only the fins to maintain equilibrium
Distance travelled	Distance travelled in straight lines within the aquarium
Mean speed	Distance travelled/time
Number of complete turns	Number of times the fish performed a complete turn
Number of crossings	Number of times the fish moved from the upper to the lower half of the aquarium or vice versa

**Table 2:** Primers designed for qPCR expression analysis in the head kidney of *Sparus aurata*, considering the genomic structure for the primers in two adjacent exons and with one large intron in the middle. Forward (F) and reverse (R) nucleotide sequences, amplicon size (bp), efficiency value (%), and R^2^ of the standard curves.

Gene	Acronym	Accession Number		Primer Sequence	Amplicon Size (bp)	E (%)	R^2^
*actb1*	actin beta 1	XM_030406939.1	F	AGCCAACAGGGAGAAGATGA	100	98	0.999
			R	ACCAGAGGCATACAGGGACA			
*eef1a*	eukaryotic elongation factor 1 alpha	AF184170.1	F	GATGGCACGGTGACAACAT	200	97	0.999
			R	AGTTCCAATACCGCCGATTT			
*star*	steroidogenic acute regulatory protein	EF640987.1	F	GAAGACCCGAACAAGACCAA	138	95	0.999
			R	ATTAGCCATCCTTTGCCTGA			
*cyp11b1*	cytochrome P450 11B, mitochondrial-like	XM_030394987.1	F	CTGCTGAAAGGCACAGTCAA	174	98	1.000
			R	CAGTGGGTCCTCAAACACCT			
*nr3c1*	glucocorticoid receptor	XM_030437675.1	F	TCTACTCGGGCTACGACAGC	181	96	0.999
			R	ATGAGGAAGAGCCAAGAGCA			
*hsd11b2*	corticosteroid 11-beta-dehydrogenase isozyme 2-like	XM_030415446.1	F	TCCTGCCCTCCTCATACAAG	118	107	0.995
			R	TAGTCCTCGCCGTAGTCCTC			
*nr3c2*	mineralocorticoid receptor	XM_030418022.1	F	CTGAAGAACCAGGCAGCATT	194	103	0.999
			R	TGGGACTCACGAAAGGTGTA			

**Table 3:** Behavioural parameters assessed in gilthead seabream juveniles exposed to different limonene concentrations. Data are the mean ± SEM of 10 fish. Two-way ANOVA, followed by Tukey’s test, was performed to assess the effect of treatment (CTRL, E, LMN20, and LMN30) over time (T0, T6, T10, and T20). Different letters indicate significant differences among treatments at each time (one-way ANOVA followed by Tukey test *p* < 0.05).

	T0				T6				T10				T20				*p*-Value (Two-Way ANOVA)		
	CTRL	E	LMN20	LMN30	CTRL	E	LMN20	LMN30	CTRL	E	LMN20	LMN30	CTRL	E	LMN20	LMN30	Time	Treatment	Interaction
Time at the bottom (s)	29.9 ± 0.1 ^a^	29.8 ± 0.2 ^ab^	25.6 ± 2.2 ^ab^	22.3 ± 3.3 ^b^	29.9 ± 0.8	29.8 ± 1.8	25.9 ± 2.2	22.5 ± 2.3	27.3 ± 1.3 ^ab^	28.8 ± 1.2 ^a^	21.8 ± 3.8 ^ab^	17 ± 3.5 ^b^	29.8 ± 0.2	30 ± 0	29.4 ± 0.5	30 ± 0	0.002	0.0006	0.167
Time leaning down (s)	16.7 ± 2.4	12.9 ± 2.9	13.8 ± 3.6	16.9 ± 3.7	7.8 ± 2.7	1.3 ± 0.6	6 ± 3.0	2.3 ± 0.9	4.5 ± 1.6	1.22 ± 0.8	4.3 ± 1.7	1.8 ± 0.7	0.7 ± 0.3	6.8 ± 3.2	1.8 ± 1.2	7.6 ± 2.8	<0.0001	0.713	0.147
Time leaning up (s)	0.0 ± 0.0	0.0 ± 0.0	0.0 ± 0.0	0.0 ± 0.0	0.0 ± 0.0	0.3 ± 0.2	0.4 ± 0.3	0.9 ± 0.3	0.1 ± 0.1	0.3 ± 0.3	4.8 ± 3.2	1.8 ± 0.6	0.1 ± 0.1	0 ± 0	0.7 ± 0.6	0 ± 0	0.070	0.144	0.206
Freezing time (s)	4.8 ± 1.8 ^a^	2.3 ± 1.2 ^ab^	0.0 ± 0.0 ^b^	0.0 ± 0.0 ^b^	17.6 ± 3.9 ^a^	4.2 ± 1.9 ^b^	2.2 ± 1.6 ^b^	3.1 ± 1.7 ^b^	13.4 ± 3.8	7.9 ± 2.7	4 ± 2.1	2.9 ± 2.2	12.2 ± 3.9	9.7 ± 3.9	2.4 ± 1.4	4.9 ± 2.2	0.001	0.001	0.252
Distance travelled (mm)	949.5 ± 251.7	1305 ± 275.2	1243.5 ± 368.9	1758.3 ± 115.8	325.5 ± 129.1 ^b^	580 ± 216.0^b^	1200.8 ± 368.0 ^ab^	1737.8 ± 416.7 ^a^	367.8 ± 147.4	510 ± 235.9	918. 5± 224.5	951 ± 295.7	181 ± 73.1 ^b^	307 ± 154.4 ^b^	728.3 ± 169.3 ^ab^	1232.2 ± 301 ^a^	0.001	0.0003	0.723
Mean speed (m/s)	0.03 ± 0.01	0.04 ± 0.01	0.04 ± 0.01	0.06 ± 0.004	0.01 ± 0.004 ^b^	0.02 ± 0.01 ^ab^	0.04 ± 0.01 ^ab^	0.05 ± 0.01 ^a^	0.01 ± 0.005	0.02 ± 0.01	0.03 ± 0.01	0.02 ± 0.01	0.01 ± 0.00 ^b^	0.01 ± 0.01 ^ab^	0.02 ± 0.01 ^ab^	0.04 ± 0.01 ^a^	0.001	0.002	0.646
Number of complete turns	2.6 ± 0.7	3.6 ± 0.6	2.2 ± 0.42	2.9 ± 0.5	0.6 ± 0.3	0.9 ± 0.3	0.7 ± 0.3	1.1 ± 0.4	0.6 ± 0.2	0.8 ± 0.4	1.0 ± 0.3	0.8 ± 0.3	0.2 ± 0.1	0.6 ± 0.3	0.6 ± 0.3	1.7 ± 0.5	<0.0001	0.096	0.391
Number of crossings	0.0 ± 0.0 ^b^	0.11 ± 0.11 ^b^	2.0 ± 0.9 ^ab^	2.9 ± 0.9 ^a^	0.11 ± 0.11 ^b^	1.5 ± 0.8 ^b^	1.6 ± 0.9 ^b^	5.5 ± 1.6 ^a^	0.6 ± 0.2 ^b^	0.0 ± 0.0 ^b^	1.2 ± 0.5 ^ab^	3.4 ± 1.1 ^a^	0.1 ± 0.1	0 ± 0	0.3 ± 0.2	0 ± 0	0.004	<0.0001	0.009

**Table 4:** Water quality parameters after 6 h transport of gilthead seabream. Water initial conditions (T0) transported fish without treatment (CTRL), with ethanol (E), or limonene at 30 µL/L (LMN30). Data are the mean ± SEM of 12 fish. The *p*-value is the result of one-way ANOVA.

	T0	CTRL	E	LMN30	*p*-Value
O_2_ (mg/L)	Saturated	3.70 ± 0.56	4.42 ± 1.23	6.27 ± 0.45	0.178
O_2_ (%)	Saturated	50.67 ± 7.62	60.80 ± 16.78	84.83 ± 6.39	0.113
pH	7.50	6.69 ± 0.03	6.74 ± 0.02	6.73 ± 0.03	0.596
Ammonium (NH_3_)	0.00	0.003 ± 0.00	0.004 ± 0.00	0.003 ± 0.00	0.667
Nitrite (NO_2_^−^ ppm)	0.00	0.28 ± 0.01	0.29 ± 0.03	0.26 ± 0.02	0.535

**Table 5:** Haematocrit and plasma and hepatic metabolite levels of gilthead seabream after 6 h transport. Non-transported fish (PRE) and transported fish without treatment (CTRL), with ethanol (E), or limonene at 30 µL/L (LMN30). Data are the mean ± SEM of 12 fish. The *p*-value is the result of one-way ANOVA.

	PRE	CTRL	E	LMN30	*p*-Value
Haematocrit (%)	33.13 ± 1.18	36.11 ± 1.05	35.52 ± 1.32	35.33 ± 0.94	0.523
Plasma					
Protein (mg/mL)	39.74 ± 1.85	43.65 ± 2.10	36.74 ± 2.19	39.42 ± 1.85	0.148
Cholesterol (mg/dL)	404.0 ± 25.3	446.3 ± 20.0	449.0 ± 22.8	398.6 ± 19.0	0.210
Liver					
Glucose (mg/g tissue)	0.92 ± 0.17	1.27± 0.13	1.19 ± 0.13	1.22 ± 0.09	0.354
Glycogen (mg/g tissue)	16.77 ± 0.89	16.82 ± 0.33	17.43 ± 0.54	18.12 ± 0.38	0.217
Lactate (mg/g tissue)	0.18 ± 0.01	0.17 ± 0.04	0.23 ± 0.03	0.24 ± 0.03	0.314
TAG (mg/g tissue)	37.32 ± 7.68	43.33 ± 4.25	47.96 ± 3.74	53.30 ± 5.26	0.201

## Data Availability

The original contributions presented in this study are included in the article. Further inquiries can be directed to the corresponding author.
